# Divergent changes in regional pulmonary filling characteristics during endotoxin-induced acute lung injury in pigs

**DOI:** 10.1186/cc9565

**Published:** 2011-03-11

**Authors:** A Aneman, S Sondergaard, A Fagerberg, H Einarsson

**Affiliations:** 1 Liverpool Hospital, Sydney, Australia; 2 Sahlgrenska University Hospital, Gothenburg, Sweden

## Introduction

Divergent regional filling characteristics of the lung may explain ventilator-induced lung injury. In this descriptive study, the potential of electrical impedance tomography (EIT) to determine progressive changes in regional filling characteristics during acute lung injury was explored.

## Methods

Endotoxin was infused during 150 minutes in 11 mechanically ventilated pigs (VC, TV 10 ml/kg, PEEP 5, RR set to normocapnia at I:E 1:2). EIT (Evaluation Kit 2; Dräger Medical) was used to monitor global and regional (four equal ventrodorsal regions of interest, ROIs 1 to 4) impedance changes at the mid-thoracic level. The tidal regional versus global impedance changes were normalized and analysed by second-degree polynomial correlation [1]. A square coefficient (*x*^2^) <0 indicates hyperinflation, >0 indicates recruitment and a value around 0 indicates homogeneous regional to global filling. Statistical evaluation was by ANOVA and Kruskal-Wallis *post-hoc *test, significance was set at *P *< 0.05.

## Results

**Figure 1:**
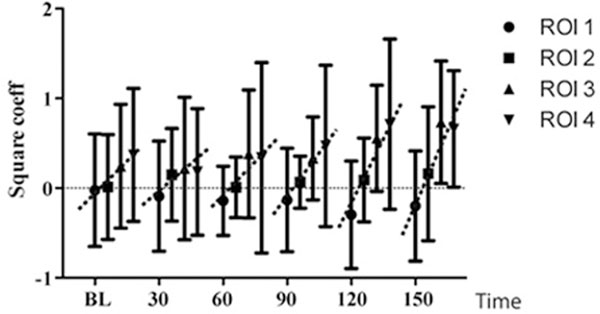


Endotoxinaemia increased the A-a O_2 _gradient and shunt, Qs/Qt, from 5.7 ± 3.6 to 33 ± 24 kPa and from 9.2 ± 2 to 27 ± 6%. Homogeneous filling in all four ROIs occurred at baseline (Figure 1) but progressively changed to hyperinflation in ROI 1 (*x*^2 ^= -0.36) and recruitment (*x*^2 ^= 0.66) in ROI 4 at 150 minutes, with ROIs 2 and 3 showing intermediate but similar changes. The *x*^2 ^gradient from ROIs 1 to 4 (dotted line) increased significantly consistent with increased regional heterogeneity comprising hyperinflation as well as recruitment.

## Conclusions

EIT can identify lung areas showing hyperinflation, recruitment or homogeneous filling, allowing ventilator settings to be adjusted to optimize pulmonary filling characteristics. Monitoring by EIT may thus potentially be used to minimize ventilator-induced lung injury.
